# Professional phagocytes are recruited for the clearance of obsolete nonprofessional phagocytes in the *Drosophila* ovary

**DOI:** 10.3389/fimmu.2024.1389674

**Published:** 2024-06-27

**Authors:** Alexandra Y. Chasse, Shruthi Bandyadka, Max C. Wertheimer, Sandy B. Serizier, Kimberly McCall

**Affiliations:** ^1^ Program in Molecular Biology, Cell Biology & Biochemistry, Boston University, Boston, MA, United States; ^2^ Program in Bioinformatics, Boston University, Boston, MA, United States; ^3^ Department of Biology, Boston University, Boston, MA, United States

**Keywords:** phagocytosis, follicle, nonprofessional phagocyte, cell death, immune-privileged, Unpaired3, hemocyte, ovary

## Abstract

Cell death is an important process in the body, as it occurs throughout every tissue during development, disease, and tissue regeneration. Phagocytes are responsible for clearing away dying cells and are typically characterized as either professional or nonprofessional phagocytes. Professional phagocytes, such as macrophages, are found in nearly every part of the body while nonprofessional phagocytes, such as epithelial cells, are found in every tissue type. However, there are organs that are considered “immune-privileged” as they have little to no immune surveillance and rely on nonprofessional phagocytes to engulf dying cells. These organs are surrounded by barriers to protect the tissue from viruses, bacteria, and perhaps even immune cells. The *Drosophila* ovary is considered immune-privileged, however the presence of hemocytes, the macrophages of *Drosophila*, around the ovary suggests they may have a potential function. Here we analyze hemocyte localization and potential functions in response to starvation-induced cell death in the ovary. Hemocytes were found to accumulate in the oviduct in the vicinity of mature eggs and follicle cell debris. Genetic ablation of hemocytes revealed that the presence of hemocytes affects oogenesis and that they phagocytose ovarian cell debris and in their absence fecundity decreases. Unpaired3, an IL-6 like cytokine, was found to be required for the recruitment of hemocytes to the oviduct to clear away obsolete follicle cells. These findings demonstrate a role for hemocytes in the ovary, providing a more thorough understanding of phagocyte communication and cell clearance in a previously thought immune-privileged organ.

## 1 Introduction

Phagocytosis is an important process responsible for the removal of billions of apoptotic cells, foreign particles, and infectious agents every day (1, 2). *Drosophila* share many genetic and physiological processes that regulate phagocytosis with mammals. Like mammals, *Drosophila* have both professional and nonprofessional phagocytes that are responsible for clearing away cell debris and dying cells. One model tissue for cell clearance is the ovary, which undergoes phagocytosis of dying germline cells via nonprofessional phagocytes, a process that is conserved in mammals (3). The *Drosophila* ovary has been considered to be immune-privileged (4–6), meaning that it lacks infiltrating immune cells, such as hemocytes – i.e. *Drosophila* macrophages – in the tissue. As such, the follicle cells of the egg chambers are responsible for clearing away germ cells when they die.

**Figure 1:**
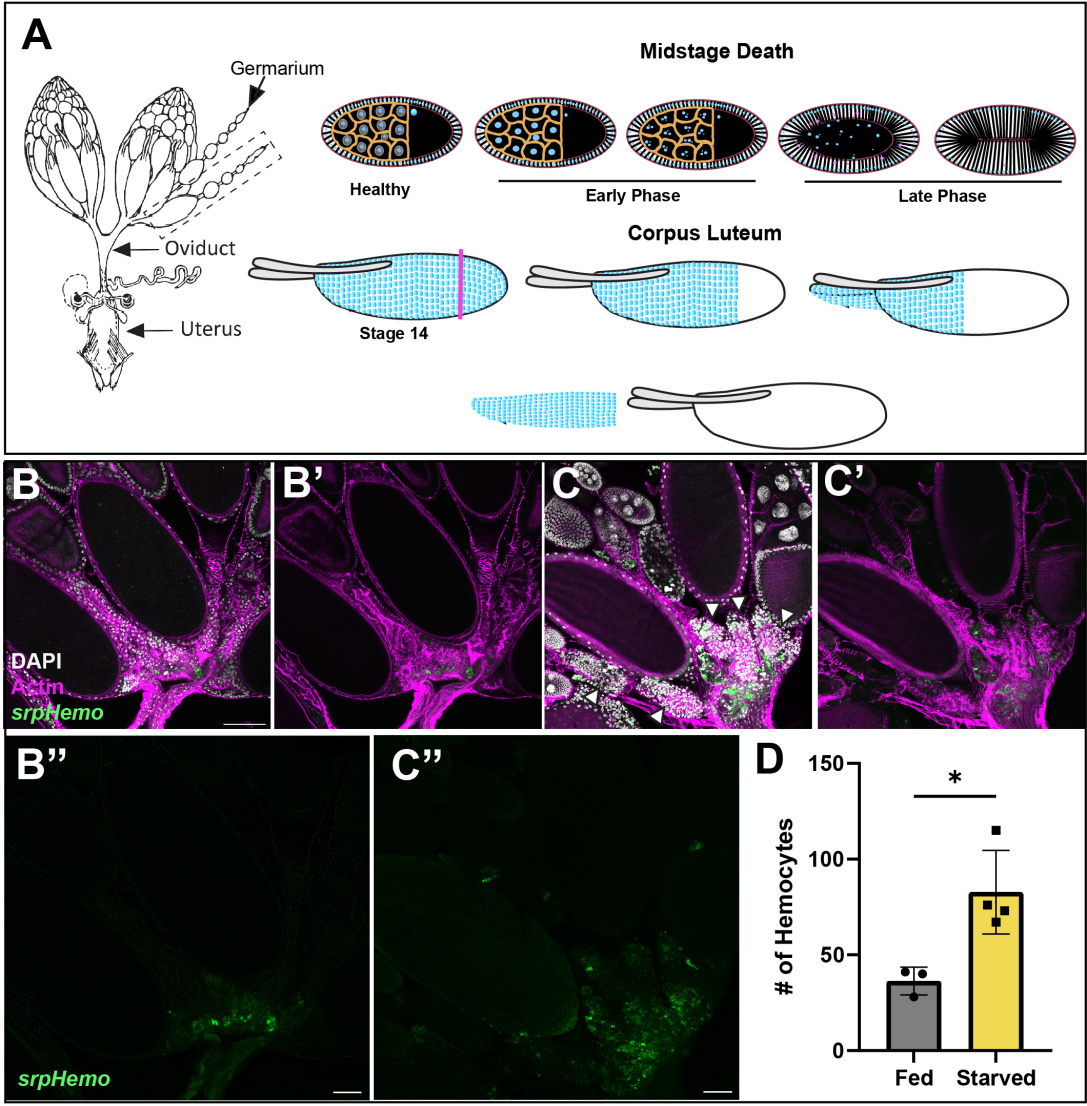
The fate of follicle cells in oogenesis. **(A)** A diagram of the female reproductive system (left) with two ovaries connected to the uterus by the oviduct. Midstage death (top) occurs when the fly is protein starved. The nurse cell nuclei (blue) condense and fragment in early death phases, while the follicle cell membrane begins to stretch inwards and engulfs the germ layer in late death phases. In corpus luteum formation (bottom), Matrix metalloproteinase2 (Mmp2) cuts the follicle cell layer at the pink axis and the follicle cell layer slides off the mature egg. **(B, C)** Hemocytes (green) are localized in the entrance to the oviduct (actin filaments (phalloidin) in purple) in both **(B)** fed and **(C)** starved flies and increased hemocyte presence around dying egg chambers (white arrowheads) by oviduct entrance. **(B’, C’)** composites without DAPI channel (white). **(B’’, C’’)** Hemocyte only channels. **(D)** Quantification of hemocytes localized in the oviduct entrance of fed and starved flies. Each data point represents two ovaries. (p-value < 0.0180). Scale bars = 50μm.

The *Drosophila* ovary is composed of egg chambers that progress through 14 stages of development. Germline cell death can occur at specific stages of development and is controlled by distinct cell death processes. Midstage death of egg chambers occurs when an egg is defective and thus needs to be cleared away or when the fly is in an unsuitable environment for laying eggs. For example, flies undergoing protein starvation trigger an increased number of midstage (stage 7–8) egg chambers to undergo apoptosis, thus providing a means to recycle nutrients to help keep the fly healthy until it can reach a more suitable environment (7, 8). The first sign of midstage cell death occurs in the germline nurse cells (NCs), whose chromatin condenses and then fragments. The surrounding somatic follicle cells (FCs) greatly enlarge and engulf the germline until it is cleared (**Figure 1A**, Etchegaray et al. (5)). An alternative cell death process occurs in late oogenesis during stages 11–14 when the egg has developed to the point where the oocyte no longer needs support from the NCs, and these are cleared away. NCs in late oogenesis die via a non-apoptotic process where stretch follicle cells surround and acidify them (9, 10). After the NCs are completely removed and the egg is mature, Matrix Metalloproteinase2 (Mmp2) cuts around the posterior end of the follicle cells, which allows the follicle cell layer to be shed (**Figure 1A**), forming the corpus luteum (11) and the egg enters the oviduct.

In both mid and late-stage death, the follicle cell layer is left behind and its clearance has not been well-studied. Possibilities for their clearance are that the follicle cells engulf each other, or that the follicle cells are pushed down into the oviduct where the oviduct cells clear them away or are expelled through the uterus. Clearance of the follicle cells is likely important in protecting the ovary from inflammation due to debris buildup (12). Inflammation could in turn affect ovary function and fertility of the fly. Interestingly, hemocytes have been noted to be around and even in the ovary since the 1970s (13) although to our knowledge, they have never been reported within an ovariole. Hemocytes, like their mammalian counterparts, are responsible for clearing dead or dying cells, pathogens, or foreign objects (14) and so it is possible that the hemocytes surrounding the ovary are involved in follicle cell clearance. Further, other studies (15) show that macrophages and epithelial cells can communicate about whether or not to engulf debris due to environmental cues, suggesting that perhaps the hemocytes could regulate the activity of the follicle cells.

Here we investigate the role that hemocytes play in the clearance of the corpus luteum and follicle cell debris left after midstage death. We found that proper progression through oogenesis is partially dependent on the presence of hemocytes despite their inability to infiltrate into the ovarioles. When hemocytes were ablated, there was persistence of follicle cell debris, suggesting that hemocytes assist in the clearance of follicle cells from both midstage dying eggs and from the corpus luteum. The cytokine Unpaired3 (Upd3) was found to be highly expressed in the follicle cells of dying midstage egg chambers, and hemocytes were localized near Unpaired3 (Upd3) positive cells in the oviduct. Further, in starved flies there was an increase in the number of hemocytes present in the oviduct and they showed activation of the JAK-STAT pathway. Mutants of *upd3* showed an increase in midstage dying egg chambers, similar to hemocyte-ablated flies, suggesting that follicle cells promote their own removal via Upd3-JAK-STAT signaling to hemocytes. These findings demonstrate a role for hemocytes in the ovary, providing a more thorough understanding of phagocyte communication and cell clearance in a previously thought immune-privileged organ.

## 2 Results

### 2.1 Hemocyte agglomeration increases in the abdomen in response to starvation

To visualize hemocytes in the fly, two different hemocyte tagged lines *Hml-Gal4.Delta*, *UAS-GFP* (hereafter *Hml* > *GFP*) and *srpHemo-mCherry* were used. To determine how hemocytes interact with the ovary, we examined ovaries from fed or starved flies expressing a hemocyte marker (*srphemo-mCherry*). Hemocytes under either condition were found to localize to the posterior of the ovary at the entrance to the oviduct. Interestingly, more hemocytes were present in the oviduct of starved flies compared to fed flies, and the hemocytes were adjacent to late phase dying egg chambers (**Figures 1B-D**). This suggests that there is a signal that is received by the hemocytes to move towards the ovaries. However, while we observed an increase in number, no hemocytes were found inside the ovariole, and were located outside the epithelial sheath. This suggests that hemocytes either play a regulatory role by communicating across the epithelial sheath, or that they follow dying egg chambers toward the oviduct, where hemocytes can enter and clear the follicle cell debris.

**Figure 2:**
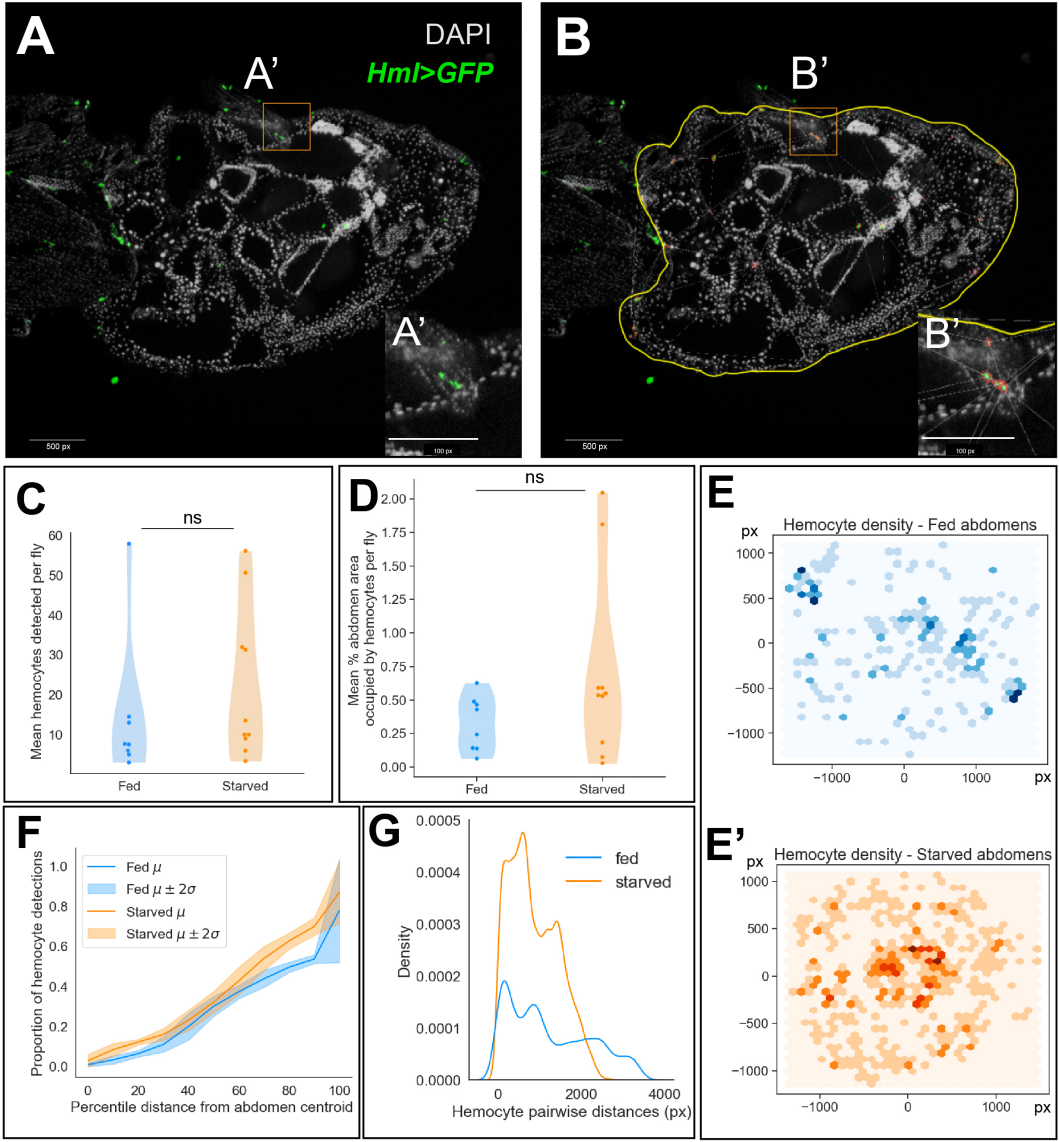
Hemocyte numbers and localization changes in response to starvation. **(A)** Representative image of a stained and sectioned abdomen (*Hml*>GFP). **(B)** Abdomen boundary is manually defined in QuPath (yellow) and hemocytes are identified (red) based on their fluorescence intensities. 1 px (pixel) = 0.207 microns x 1 micron. **(C)** Distribution of the number of hemocytes detected averaged across abdomen sections per fly in well-fed and starved experimental conditions (two-sided Mann-Whitney U-test, p-value = 0.28). **(D)** Distribution of the percentage of abdomen area occupied by hemocytes averaged across abdomen sections per fly (two-sided Mann-Whitney U-test, p-value = 0.20). **(E, E’)** 2D histogram of hemocyte cartesian coordinates. Darker regions indicate a higher number of hemocyte detections across replicates. **(F)** Empirical cumulative distribution function (ECDF) of the proportion of hemocyte detections at percentile distances away from the abdomen centroid. Mean ECDF of fed and starved replicates are indicated by dark lines and the 95% confidence intervals are indicated by the lighter color bands **(G)** Histogram of pairwise Euclidean distances between hemocytes in well-fed and starved abdomens.

To determine if hemocytes respond when cell death is induced in the ovary, we fed flies a rich diet with yeast paste and then induced protein starvation using fruit juice agar. To maintain interactions between the hemocytes and ovary, *Hml>GFP* females were frozen, sectioned, and stained to determine where hemocytes were localized (**Figure 2A**). To determine if hemocyte localization changed following starvation, we used a semi-automated method to identify hemocytes in the stained abdomen sections by performing image segmentation (**Figure 2B**), followed by statistical inference on the number and spatial distribution of hemocyte detections.

We chose abdomen sections of comparable area in both fed and starved groups for quantifying hemocyte density and localization (**Supplementary Figure 1A**). We observed no significant difference in the average number of hemocytes detected across sections between the experimental groups (**Figure 2C**). Further, we observed no significant difference in the percentage of abdomen area occupied by hemocytes, averaged across sections (**Figure 2D**). Next, we wondered whether hemocytes were differentially localized to specific niches within the fed and starved abdominal cavities. We visualized the cartesian coordinates of hemocyte detections across replicate sections as a bivariate histogram (**Figures 2E–E’**). This suggested that the hemocytes were distributed sparsely, closer to the periphery of the abdomen in well-fed flies. However, in the abdomens of starved flies, we observed agglomeration of hemocytes closer to the abdomen centroid. As the ovary is more centrally localized in the abdomen, taking up around 60% of the abdomen cavity, we sought to determine whether the proportion of hemocytes present throughout the abdomen differed significantly between fed and starved flies. To quantify this observation, we visualized the proportion of hemocyte detections at increasing percentile distances away from the abdomen centroid as a cumulative distribution. Starting from the abdominal region at 10^th^ percentile distance away from the centroid, we observed that starved abdomens contained a higher proportion of hemocytes on average (**Supplementary Figure 1B**, **Figure 2F**).

Finally, we also determined whether the hemocytes localize closely together in starved abdomens compared to well-fed controls using two complementary methods. First, we observed that pairwise distances between hemocytes in starved abdomens tended to be shorter compared to fed abdomens (**Figure 2G**). Using the Delaunay triangulation (16) method implemented in QuPath, we obtained the mean triangle area formed between hemocyte neighbors as a proportion of the abdomen area. Starved abdomens contained more hemocytes with 4–10 neighbors each and therefore the number of triangles constructed between them were higher in number and the mean triangle area tended to be smaller compared to the well-fed controls, indicating that the hemocytes were clustered closer together in starved abdomens (**Supplementary Figures 1C, D**). Next, we implemented the nearest-neighbors distribution G-function (17), which is defined as the cumulative distribution function (CDF) of nearest-neighbor distances. We visualized the mean empirical CDF of the distances of 5 nearest neighbors for each hemocyte across sections in both well-fed and starved abdomens. This indicated that the nearest hemocytes for any given hemocyte tended to be closer in starved abdomens, compared to their well-fed controls (**Supplementary Figures 1E–G**).

Our analysis therefore indicates that hemocytes respond to starvation-induced cell death signaling and that the response is orchestrated by their agglomeration at specific sites within the abdomen which could enable them to interact with each other and mount a coordinated response where needed.

### 2.2 Hemocyte ablation results in ovarian debris persistence and decreases lifespan

**Figure 3:**
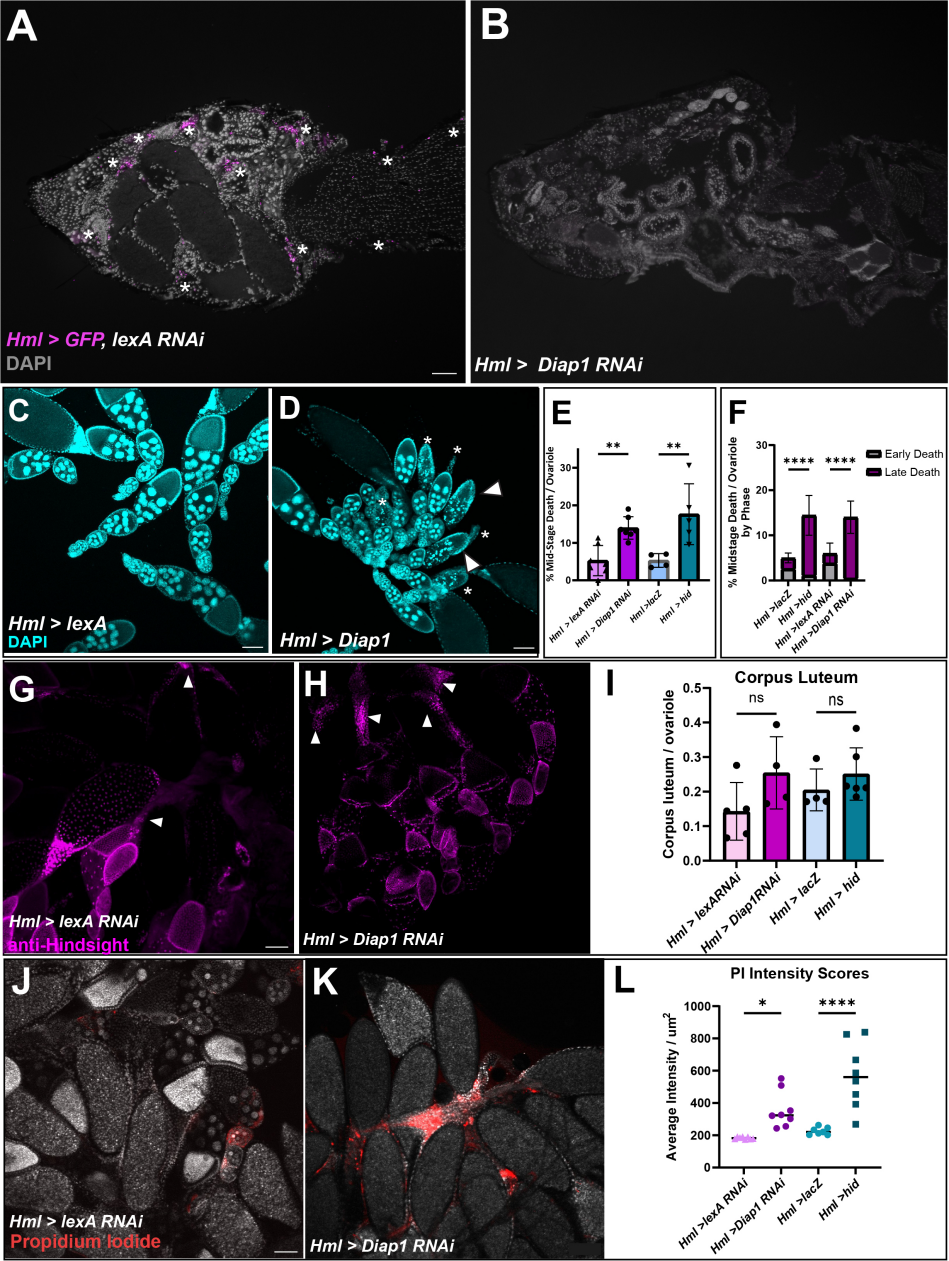
Hemocyte ablation results in defective oogenesis and reproductive defects. **(A, B)** Cryosections of *Hml*>*lexA RNAi*, *GFP* adult female with hemocytes in magenta (white asterisks). **(B)** *Hml* > *Diap1 RNAi*, *GFP* adult female with no hemocytes present. **(C)** *Hml > lexA RNAi* control egg chambers and **(D)** *Hml > Diap1 RNAi* egg chambers stained with DAPI (cyan). White arrowhead marks early degenerating egg chambers and asterisks mark late phase degenerating egg chambers. **(E)** Quantification of midstage death in controls (*Hml > lacZ* and *Hml > lexA RNAi*) and hemocyte-ablated (*Hml > hid* and *Hml > Diap1 RNAi*) fed flies, n = 7–10 flies per replicate with total of > 40 females per genotype. **(F)** Dying egg chambers from controls and ablated flies were identified by death phase (early and late). Ordinary One-Way ANOVA determined that ablated flies had a highly significant increase in the number of late stage dying egg chambers. **(G)** Representative image of *Hml > lexA* RNAi with anti-Hindsight staining the corpus luteum (in magenta). **(H)** Representative image of *Hml > Diap1* RNAi ovaries with anti-Hindsight staining the corpus luteum (in magenta). **(I)** Quantification of corpus luteum retention, n = 7–10 females per replicate with a total of > 30 females per genotype. **(J)** Representative image of *Hml > lexA RNAi* egg chambers stained with Propidium Iodide (red). **(K)** Representative image of *Hml > Diap1 RNAi* egg chambers stained with Propidium Iodide (red). **(L)** Quantification of PI fluorescence, n = 8 females per genotype. (* p-value < 0.02,** p-value < 0.002, **** p-value < 0.0001) Scale bars = 100µm.

To determine whether hemocytes affect oogenesis, hemocytes were genetically ablated using the *UAS*-*Gal4* system. *Hml-Gal4* flies were crossed with either *UAS-Diap1 RNAi* or *UAS-hid* to induce apoptosis of hemocytes (18) or to *UAS-lexA RNAi* or *UAS-lacZ* for controls. To ensure that hemocytes were ablated, flies also carrying *UAS-GFP* were cryosectioned and imaged. As shown in **Figures 3A, B**, *Hml>GFP, lexA RNAi* flies had hemocytes present while *Hml>GFP, Diap1 RNAi* flies had no detectable hemocytes. Since these flies now lacked a major component of the innate immune system, we tested whether their lifespan was impacted. Hemocyte-ablated flies had shortened lifespans compared to control flies and began dying as early as day 6. The 50% survival rate for both *Hml>hid* and *Hml>Diap1 RNAi* flies fell to days 35 and 60 respectively, whereas control flies averaged about 65 days (**Supplementary Figure 2**). However, hemocyte-ablated flies lived on average around 40 days, which was sufficiently long enough for analysis on the effects on oogenesis.

To determine whether the absence of hemocytes affected proper oogenesis, we dissected the ovaries of fed or starved young females (5–10 days) and used DAPI to visualize nuclei of egg chambers. We found that fed hemocyte-ablated flies had an increased number of midstage dying egg chambers compared to fed control flies (**Supplementary Figure 3A**). The number of dying egg chambers in the fed ablated flies was more than double the amount in the control flies (**Figures 3C–E**), and comparable to the amount found in starved flies (**Supplementary Figure 3A**). As egg chambers undergo midstage death, the nurse cells are broken down in the early phases of death and engulfed in the late phases of death, leaving behind mainly follicle cells by phases 4 and 5 (5). Interestingly, when we scored the dying egg chambers by phase of death (**Figure 1A**), the ablated flies had a higher proportion of late phase egg chambers whereas the control flies contained more early phase egg chambers (**Figure 3F**), suggesting that the dying egg chambers were persisting in the ovary and not being cleared efficiently. Ovaries were also stained with anti-Hindsight to quantify the amount of corpus luteum present (11). While there was no significant difference between the controls and experimental ovaries, there was a slight trend where ablated flies had a higher ratio of corpus luteum present compared to control flies (**Figures 3G-I**), which may suggest that clearance of corpus luteum could be impacted. These findings suggest that hemocytes are required for the efficient removal of dying mid-stage egg chambers and may assist in corpus luteum clearance from late-stage egg chambers.

### 2.3 Hemocyte ablation leads to a build-up in cell debris that becomes necrotic and affects fecundity

We found that the hemocyte-ablated flies showed an increase in FC debris compared to the control, which could lead to secondary necrosis. To determine if the hemocyte-ablated flies showed an increase in necrosis, we used propidium iodide to stain necrotic cells (19). The intensity of the PI stain in each ovary was measured using ImageJ and ablated flies showed a 1.5–3-fold increase of fluorescence intensity per µm^2^ compared to the control flies, demonstrating that necrosis occurs in the egg chamber debris that builds up when hemocytes are not present (**Figures 3J–L**).

Given the excessive debris accumulation in the ovary, we sought to determine if hemocyte ablation resulted in decreased fecundity by counting the number of eggs laid. *Hml>hid* flies showed a significant decrease in fecundity (**Supplementary Figure 3B**). *Hml>Diap1 RNAi* flies did not significantly differ from its control but did have a downward trend when compared to the control. These results suggest that the absence of hemocytes results in a decrease in the ability to lay eggs perhaps due to the cell debris persistence.

### 2.4 Hemocytes engulf ovarian follicle cell remains

**Figure 4:**
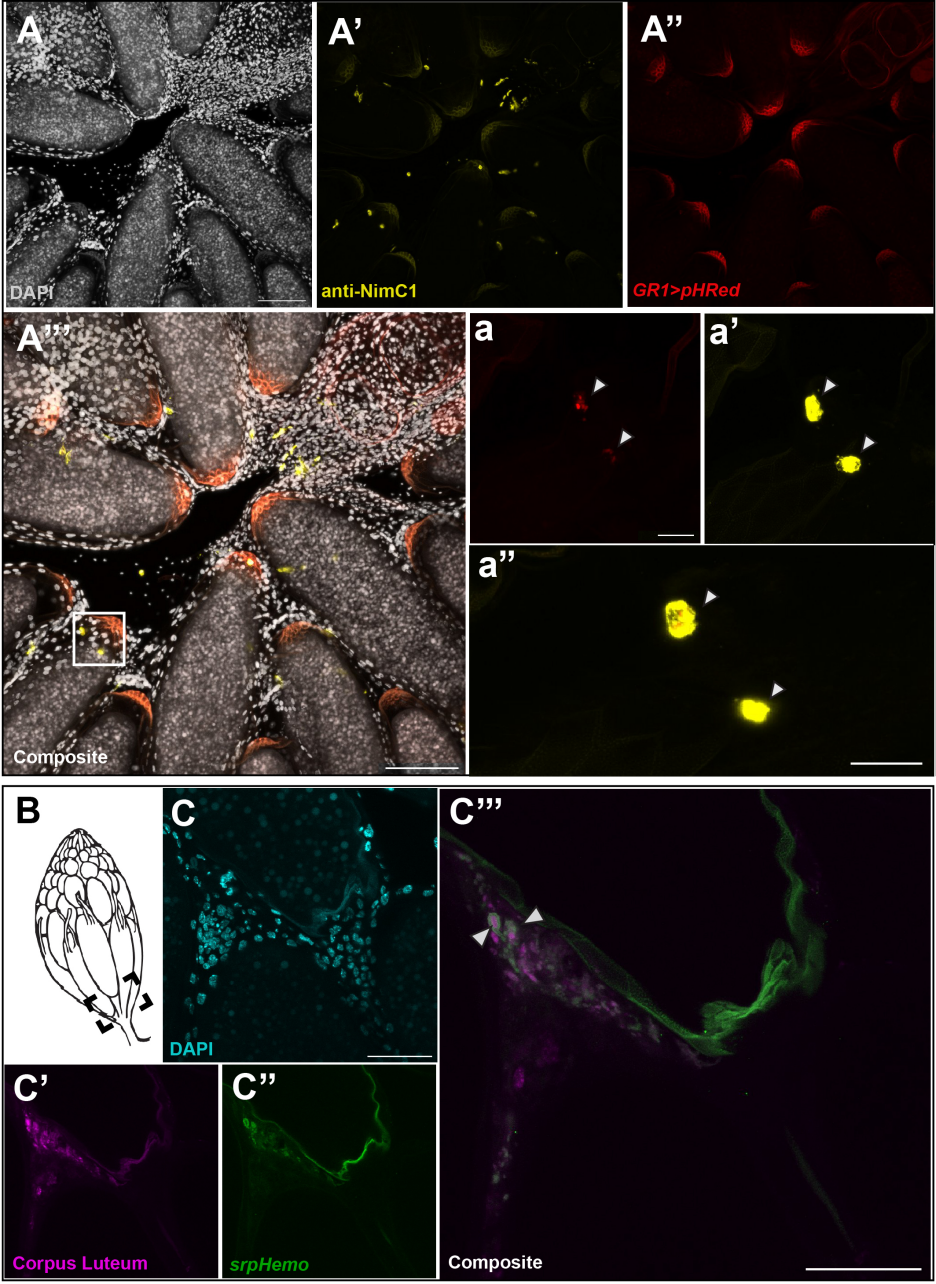
Hemocytes are involved in the engulfment of cell debris. **(A)** DAPI staining of entrance to oviduct. **(A’)** anti-NimC1 staining. **(A’’)** pHRed expressed with the follicle cell driver *GR1-Gal4*. **(A’’’)** Composite of NimC1 (yellow) and pHRed channels, with a white box marking the zoom window. Scale bar =100μm. **(a)** pHRed under *GR1-Gal4 driver*. **(a’)** NimC1 antibody staining, merge of 13 5 μm z stacks. **(a”)** Composite of pHRed and NimC1 antibody channels, z = 3 in order to visualize pHRed signaling. Colocalization is marked by white arrows. Scale bar = 20μm. **(B)** Diagram of ovary with dotted box indicating imaging area. **(C’)** DAPI in oviduct region. **(C’’)** anti-Hindsight staining of the corpus luteum (magenta). **(C’’’)** Hemocyte tagged line *srpHemo-mCherry* (green). **(C’’’’)** Composite of srpHemo and Hindsight channels; white arrowhead marks hemocyte engulfment of corpus luteum. Scale bars = 50μm.

The increased number of late phase midstage dying egg chambers in the ovary when hemocytes were ablated suggested that hemocytes were required for clearance of follicle cells that were no longer needed. To visualize follicle cell clearance, we expressed a pH sensitive marker (*UAS-pHRed-CAAX*, 10) in follicle cells using midstage follicle cell driver *GR1-Gal4*. pHRed-CAAX only fluoresces in a low pH environment and can be used to detect phagocytosis by another cell. Using both fixed tissue and live imaging, we detected expression of pHRed at the entrance of the oviduct that colocalized with hemocytes (**Figures 4A–A’’’**). Hemocytes were labeled with an antibody against NimrodC1 (NimC1) (20), a highly expressed transmembrane receptor in hemocytes (21). Colocalization of pHRed with NimC1 staining (**Figure 4A’’’**) was closely examined and a zoom in on hemocytes located in the calyx of the oviduct is shown in **Figures 4A–A”**. The corresponding hemocytes are marked by a white box in **Figure 4A”’**. Colocalization of pHRed with hemocytes in panel 4a” demonstrates that hemocytes engulf midstage dying follicle cells before breaking them down in the phagosome. It is important to note that the posterior region of egg chambers is autofluorescent and non-specific staining is seen in both the yellow and red channels. There was no evidence that follicle cells engulf each other, as no pHRed fluorescence was detected in late phase follicle cells before entering the oviduct (**Supplementary Figure 4**).

Similarly, the slight increase in corpus luteum remaining in the ablated flies compared to the control flies suggested that hemocytes may also be involved in the engulfment of the corpus luteum. To examine whether hemocytes are involved in corpus luteum engulfment, we dissected the ovaries of *srpHemo-mCherry* females and stained with anti-Hindsight to detect the corpus luteum (11). As the corpus lutea are deposited at the entrance of the oviduct (**Figure 4B**), the area was carefully examined, and it was observed that some hemocytes had engulfed Hindsight-positive follicle cells from the corpus luteum in the entrance of the oviduct while other hemocytes were localized near other Hindsight-positive cells (**Figures 4C–C’’’**). However, the frequency of hemocytes engulfing the corpus luteum was low, suggesting that hemocytes assist in the clearance of the corpus luteum, though the corpus luteum may be cleared in another fashion.

### 2.5 Upd3 is expressed in the follicle cells of dying egg chambers and promotes midstage death clearance

We next wanted to determine what signals the dying follicle cells could be releasing to attract hemocytes to the oviduct. One strong candidate was the JAK/STAT (Janus kinases and signal transducer and activator of transcription proteins) signaling pathway which has been shown to be activated in hemocytes (22) Upd3 is a cytokine and JAK/STAT pathway ligand known for intercellular signaling which mediates immune responses (23) and can be secreted by both immune and nonimmune cells. As *Drosophila* have a limited number of hormones and cytokines, Upd3 plays numerous roles including aseptic and septic injury (24, 25), parasitoid wasp infection (26) and bacterial infections (27). Upd3 also regulates cell survival, proliferation, and migration, thus promoting metastasis in cancers (28–30), and, when dysregulated, can result in inflammation and autoimmune diseases (31, 32). Ammeux (33) observed that a *upd3* reporter was expressed in the follicle cells of midstage dying egg chambers, with the signal increasing in intensity as the egg chamber moves into the late phases of death. Taken together, this presented as strong evidence for Upd3 as a candidate for follicle cell to hemocyte signaling.

**Figure 5:**
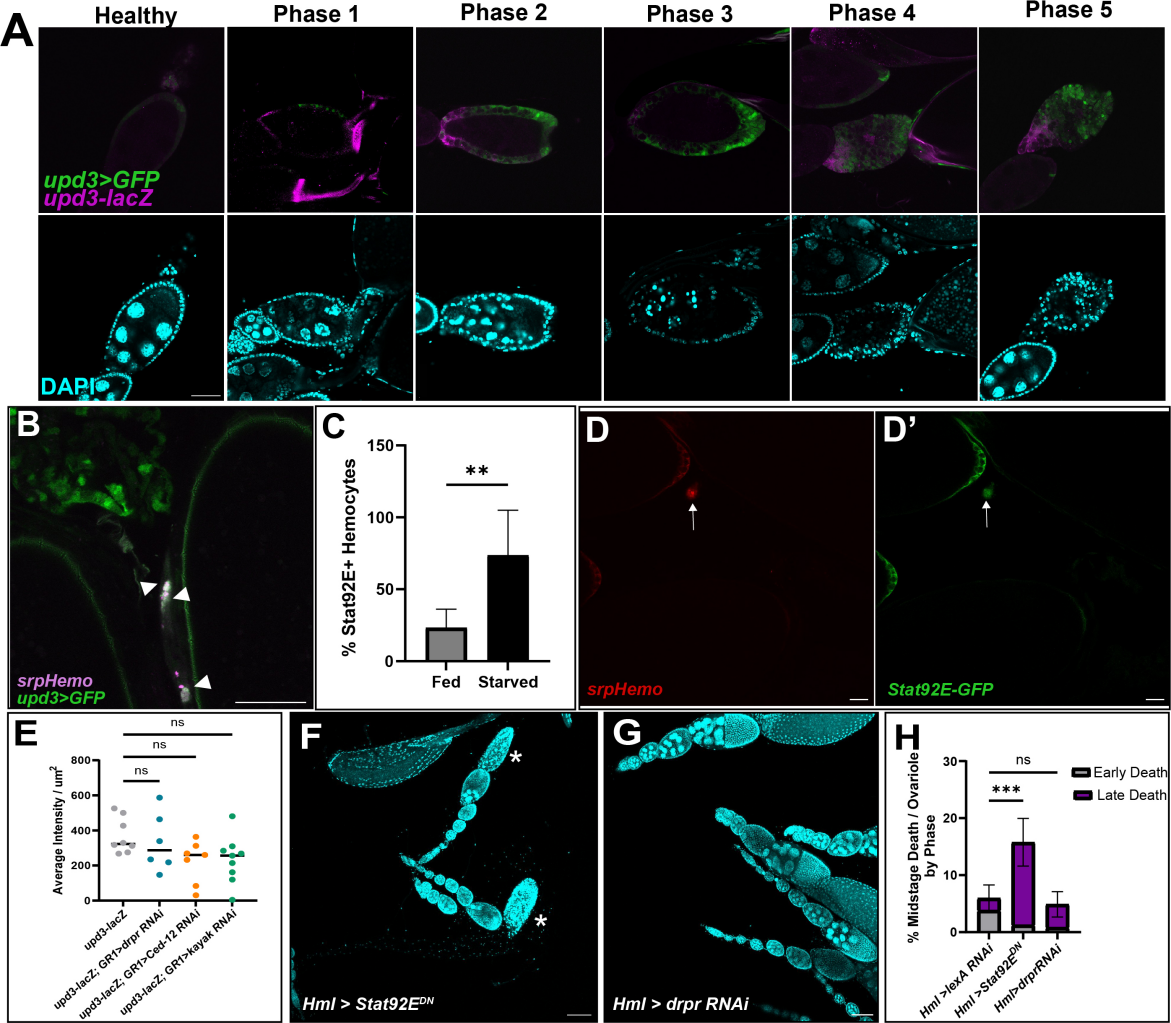
Upd3 is expressed in the follicle cells of dying egg chambers. **(A)** Egg chambers are ordered by death phase, starting with Healthy (left) and ending with Phase 5 (right). Top panels are *upd3>GFP* (green) and *upd3-lacZ* (magenta) co-expression. Bottom panels are DAPI (cyan) of corresponding egg from above image. Scale bars = 50μm. **(B)** Hemocytes (magenta) are localized near and engulfing Upd3 positive cells (green, white arrowheads). Scale bar = 50μm. **(C)** Percentage of STAT+ hemocytes in the oviduct of fed or starved females, n = 7 females per condition. Unpaired t-test determined that the number of STAT+ hemocytes increased in starved females (** p-value = 0.002). **(D, D’)** Representative image of STAT+ hemocytes. **(D)** *srpHemo-mCherry* channel. **(D’)** Stat92E-GFP channel. White arrows indicate hemocyte with Stat92E-GFP expression overlap. Scale bar = 20μm. **(E)** *upd3-lacZ* expression was quantified in knockdowns of several phagocytic genes (*draper RNAi* #67034). Average intensity per μm^2^ were compared across genotype. Ordinary One-Way ANOVA reveals no significant difference (ns, p-value > 0.05). **(F)** Representative image of fed *Hml>Stat92E^DN^* ovaries with arrowheads indicating early phase dying egg chambers and asterisks indicating late phase dying egg chambers. **(G)** Representative image of fed *Hml>draper RNAi* (#36732) ovaries with arrowheads indicating early phase dying egg chambers and asterisks indicating late phase dying egg chambers. Scale bars = 100μm. **(H)** Quantification of midstage death phases of both *Hml > Stat92E^DN^* and *Hml > draper RNAi* (#36732) ovaries compared to *Hml > lexA RNAi* control ovaries, n = 7–10 females per replicate and > 14 females per genotype total. 2way ANOVA determined that the percentage of late phase dying egg chambers increased significantly in *Hml > Stat92E^DN^* compared to control, but that *Hml > draper RNAi* was not significant (*** p-value < 0.001, ns p-value > 0.05).

We visualized *upd3* expression with two different reporters: *upd3-lacZ* and *upd3-Gal4, UAS-GFP* (*upd3>GFP*, 34). The *upd3-lacZ* reporter was expressed in follicle cells early during egg chamber death starting at the anterior follicle cells. This expression continued towards the posterior follicle cells as death progressed; however, the signal was weaker during the later death phases. *upd3>GFP* expression began later at the mid-death phases and remained strongly expressed through the late death phases (**Figure 5A**). There were spatial differences between the reporters which are likely due to differences in the length of regulatory sequences, vector differences or position effects (34). However, both *upd3* reporters became activated in engulfing follicle cells during mid-stage egg chamber death, suggesting that Upd3 could be a signal to the germline, the follicle cells, or cells outside the ovary, such as hemocytes.

We next sought to determine whether hemocytes were responding to the *upd3*-expressing follicle cells. Using *upd3>GFP; srpHemo-mCherry* flies, hemocytes were found to cluster around the *upd3>GFP* positive follicle cells once they entered the oviduct (**Figure 5B**). This suggests that Upd3 may be the “Find Me” signal that follicle cells express when they need to be cleared. We also used a *Stat92E-GFP* reporter and determined that there was an increase in Stat92E+ hemocytes present in the oviduct entrance of starved flies compared to fed flies (**Figures 5C, D**).

As we had found that Upd3 may be the recruiting factor for hemocytes, we aimed to determine the pathway that activates *upd3* expression in engulfing follicle cells. We have previously identified a number of signaling pathways that are involved in *Drosophila* cell death signaling in midstage egg chamber death (35). The phagocytic receptor Draper (Drpr) is expressed on the membranes of follicle cells and is required for phagocytosis of the dying germline (5). Signaling by Draper leads to downstream activation of the JNK pathway as well as the reorganization of actin in the follicle cells to allow for engulfment of the dying cell. We examined knockdown lines of *draper*, as well as another engulfment gene *Ced-12*, and the JNK pathway gene *kayak* for effects on *upd3* expression but it was not significantly different than controls (**Figure 5E**), thus the pathway leading to upregulation of *upd3* in engulfing follicle cells remains unknown.

To determine if JAK/STAT activation in hemocytes was involved in egg chamber clearance, we expressed a dominant negative form of Stat92E in hemocytes (**Figure 5F**). We found an increase in the percentage of late phase midstage dying egg chambers in fed females (**Figure 5H**), similar to hemocyte ablation. This similar phenotype suggests that hemocytes are necessary for proper ovary homeostasis and that JAK/STAT activation is required for proper clearance of the midstage dying egg chambers. Interestingly, when we knocked down *draper* in hemocytes using RNAi, we did not see an increase in either the number of midstage dying egg chambers in fed females (**Figures 5G, H**). This indicates that hemocytes do not require *draper* to clear away obsolete follicle cells from the ovary.

### 2.6 Upd3 expression in follicle cells is required for clearance of late phase dying egg chambers and hemocyte recruitment to the oviduct

**Figure 6:**
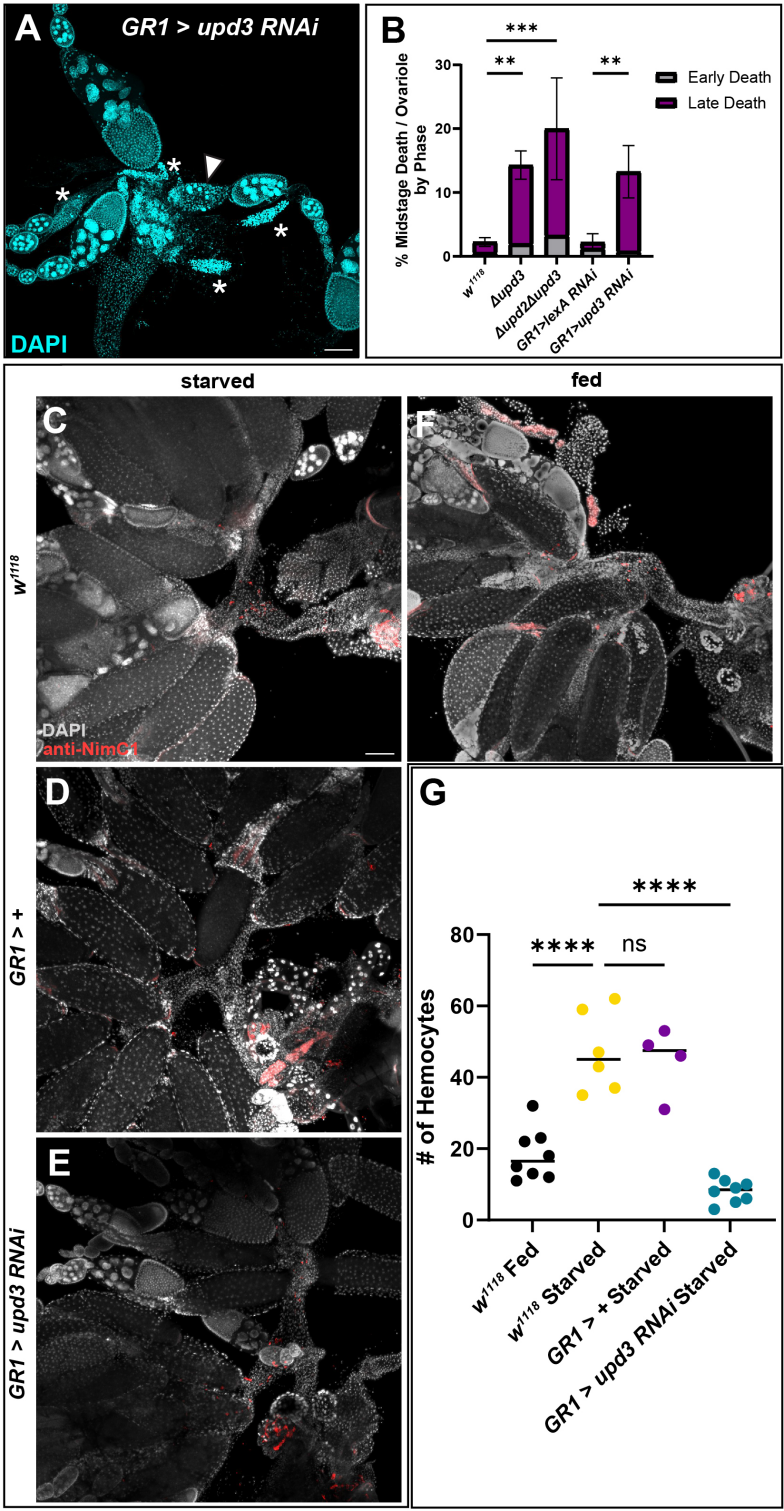
Absence of *upd3* expression in dying follicle cells results in midstage death persistence and decreased hemocyte numbers. **(A)** Follicle cell knockdown of *upd3* results in an increased number of late phase dying egg chambers (asterisks). **(B)** Quantification of dying egg chambers by death phase (** p-value of < 0.007, *** p-value of < 0.003), n = 10–15 females per replicate with a total of > 25 females per genotype. **(C)** *w^1118^* starved flies with hemocytes labeled in red. **(D)** *GR1-Gal4* starved ovaries. **(E)** *GR1>upd3 RNAi* starved ovaries. **(F)** *w^1118^* fed ovaries. **(G)** Hemocyte recruitment to the oviduct was quantified in fed and starved conditions in both *w^1118^* and knockdown flies. Each data point indicates an individual female with n = 4–8 females per genotype and condition, with >20 ovarioles scored per female. Starved *w^1118^* flies had a significant increase in the number of hemocytes present in the oviduct compared to fed *w^1118^* flies, however starved *upd3* knockdown flies had a significant decrease in the number of hemocytes present in the oviduct (**** p-value < 0.0001, ns p-value > 0.05).

Given that *upd3* was highly expressed in the FCs of dying mid-stage egg chambers, we examined germline death progression and engulfment during midstage death in *Δupd2Δupd3* and *Δupd3* mutants and found that the events progressed similar to controls (**Supplementary Figure 5A**). Although *upd2* has been shown to be differentially expressed (36), downstream signaling events are similar between the two ligands (37), and therefore we included the double mutant. We next sought to determine if *upd3* was needed for follicle cell clearance after the completion of nurse cell death and engulfment. The ovaries of *Δupd2Δupd3* and *Δupd3* mutants were dissected and stained with DAPI and dying egg chambers were quantified. The mutants showed a higher rate of midstage death in fed mutants compared to fed *w^1118^* control flies (**Figure 6B**). To determine if this phenotype was due to *upd3* expression in follicle cells, we knocked down *upd3* specifically in follicle cells using the *GR1-Gal4* driver. *GR1>upd3 RNAi* ovaries were dissected and stained with DAPI, and it was seen that, similarly to the mutants, the *upd3* knockdown flies had an increase in midstage death compared to *GR1>lexA RNAi* flies. When the death phases were quantified, it was found that there was a higher frequency of late phase dying egg chambers compared to controls (**Figures 6A, B**), similar to what was observed with hemocyte ablation.

To determine if *upd3* affected hemocyte recruitment, we used the NimC1 antibody to quantify the number of hemocytes in the ovary by the oviduct entrance (**Figures 6C–G**). Control females from both fed and starved conditions were examined and it was determined that hemocytes were recruited to the entrance of the oviduct during starvation in higher numbers compared to the fed females (**Figures 1D**, **6C, F–G**, **Supplementary Figures 6A, B**). To determine if hemocytes responded to *upd3* expression in follicle cells, *upd3* was knocked down using RNAi specifically in follicle cells. Hemocytes within the main oviduct were not included in the quantification, and only hemocytes at the entrance of the oviduct where the ovaries meet the oviduct were counted. It was observed that hemocyte localization within the oviduct was significantly decreased in starved *upd3 RNAi* flies compared to both starved *w^1118^* and *GR1-Gal4*>+ flies (**Figures 6C-E**, **Supplementary Figure 6**) and had fewer hemocytes present compared to *w^1118^* fed females (**Figures 6F-G**, **Supplementary Figure 6B**).

## 3 Discussion

Here we examined the role of hemocytes in the *Drosophila* ovary, an organ previously thought to have no hemocyte oversight. By using several hemocyte markers, we were able to tag hemocytes and determine their patterning in fed and starved flies. We saw that starved flies had a larger number of hemocytes recruited to the oviduct and localized more centrally in the abdomen compared to fed flies. This suggests that there is hemocyte recruitment when flies are protein starved.

To determine whether the presence of hemocytes affected oogenesis, we genetically ablated hemocytes by knocking down *Diap1* or overexpressing *hid*. Ablated flies fed with yeast paste showed an increase in midstage death compared to controls, however this increase was specifically due to late phase dying egg chambers (**Figure 3F**), suggesting that there is a delay in the clearance of egg chamber remnants compared to controls. It was also observed that the ratio of corpus luteum present increased in ablated flies compared to control flies, although not significantly. This suggests that while hemocytes may contribute to corpus luteum clean-up, there is another means of follicle cell removal such as oviduct cells (38). As there was a buildup in debris, we speculated that this debris from both midstage and corpus luteum was not being cleared correctly and may undergo secondary necrosis, which could damage the tissue due to its pro-inflammatory consequences.

Propidium iodide (PI) staining for necrotic cells revealed an increase in PI staining in the ablated flies compared to control, suggesting that in the absence of hemocytes, dying cells were not cleared. As necrotic cells are harmful to surrounding tissues, we sought to determine whether there was an effect on fecundity and found that *Hml>hid* flies showed a significant decrease in fecundity compared to the control. Survival assays also revealed a decrease in average lifespan in ablated flies compared to control flies, suggesting that inflammation caused by cell debris persistence, or necrotic cells could be a contributing factor to both fecundity and lifespan. The *Hml>hid* flies’ fecundity and survival was more greatly affected compared to *Hml>Diap1 RNAi* flies which may be due to the amount of inflammation, as the *Diap1 RNAi* flies had lower amounts of PI stain compared to the *hid^OE^* flies. The lack of clearance demonstrated by the increase in late phase midstage egg chambers and the resulting necrosis suggests that inflammation may be occurring in the ovary. As we only aged the flies to day 3 and dissected by day 10 before using them for experiments, it is important to note that we are most likely seeing the effects of acute inflammation rather than chronic. Both the survival assay and the fecundity assay may show some effects of chronic inflammation as well (39–41). The effects of chronic low inflammation versus chronic high inflammation on oogenesis and fertility may be revealed through further study.

As the fate of follicle cells for both midstage death and corpus luteum clearance was unknown, we examined whether hemocytes were involved in their clearance. Follicle cells were tagged with a pH-sensitive fluorescent protein and crossed into a hemocyte reporter line. Cells that were pHRed positive colocalized with hemocytes in the entrance to the oviduct, demonstrating that hemocytes engulf follicle cell debris from midstage dying cells. Engulfment of corpus luteum follicle cells by hemocytes was also observed, however since corpus luteum clearance wasn’t severely impacted, hemocytes may not be the main force for this clearance. Thus, hemocytes may be specifically recruited to the ovary during starvation to cope with the increase in cell death.

Upd3 is a pro-inflammatory IL-6 like cytokine that acts as a ligand in the JAK/STAT pathway, a highly conserved pathway that plays an important role in development, immune responses, hematopoiesis, and cancer throughout evolution (25, 26, 36, 41–43). *upd3* is highly expressed in the follicle cells of dying egg chambers (33, **Figure 5**), however there were no engulfment defects in follicle cells or increased egg chamber death observed in *upd3* mutants when starved (**Supplementary Figure 5A**). The number of Stat92E+ hemocytes increased in the oviduct of starved flies compared to fed flies, suggesting that *upd3* expressed in follicle cells was recruiting the hemocytes. Expression of a dominant negative form of *Stat92E* in hemocytes resulted in a similar phenotype to hemocyte-ablated ovaries in fed females, suggesting that JAK/STAT activation in hemocytes is necessary for proper follicle cell clearance and ovary homeostasis. When *draper* was knocked down in hemocytes, there was no increase in the number of midstage dying egg chambers, suggesting that hemocytes may be able to either compensate with other phagocytic receptors such as Eater or Nimrod, or may not depend on Draper as comprehensively as nonprofessional phagocytes do. Future experiments knocking down these phagocytic genes both separately and together are needed to determine which phagocytic receptor(s) is required for follicle cell cleanup. Alternatively, JAK/STAT signaling may control non-phagocytic mechanisms that signal to the ovary and affect egg chamber survival. These results taken together suggest that Upd3 expressed in follicle cells activates the JAK/STAT pathway in hemocytes. As JAK/STAT is a major cell communication pathway, it is likely that the binding of Upd3 to the Dome receptor is one of the ways that hemocytes and follicle cells communicate to coordinate cleanup of dying follicle cells. As it is known that the JAK/STAT pathway and other signaling pathways, such as NF-κB and Notch, are often linked (43, 44), further studies are needed to determine whether crosstalk between different signaling pathways occurs during phagocytosis in the ovary.

We found an increase in the number of midstage dying egg chambers in *upd3* mutants compared to controls. This phenotype was similar to that seen in hemocyte-ablated flies and suggests that Upd3 from follicle cells signals to recruit hemocytes to the oviduct to facilitate the clearance of obsolete follicle cells, especially as there was a higher ratio of late phase dying to early phase dying egg chambers. Hemocytes were observed to colocalize with *upd3*+ cells suggesting that hemocytes engulf *upd3*+ follicle cells. Knocking down *upd3* in follicle cells led to a significant decrease in hemocyte recruitment, resulting in hemocyte numbers in the oviduct that were lower than those of fed control flies (**Figure 6G**). These data suggest that Upd3 is an important signaling molecule between follicle cells and hemocytes.

Our study demonstrates a role for hemocytes in the previously thought immune privileged ovary and offers new evidence of communication between professional and nonprofessional phagocytes. While the presence of hemocytes in the ovary is not fully understood, one role may be to clear the follicle cells (nonprofessional phagocytes) once they become obsolete. Mammalian models have suggested a similar role for macrophages. Developing oocytes are enclosed in primordial follicles with macrophages found in the theca layer of these follicles. Recent studies have established that the presence of macrophages in the ovarian tissue is essential for the maintenance of growing follicles and that macrophages participate in the regulation of follicular growth (45). Ablation of these macrophages resulted in hemorrhaging and widespread cell death in the ovary (46), similar to the phenotype we found in flies when hemocytes were ablated. Very few follicles achieve ovulation, and 99% of follicles in the mammalian ovary undergo atresia, a process similar to midstage death in *Drosophila.* Studies in mice show that macrophages are recruited to the follicles undergoing atresia (47), a phenomenon that was also shown in our studies in *Drosophila* (**Figure 6G**). These findings suggest that macrophage recruitment to and communication with somatic ovary cells is an evolutionarily conserved process, required for ovary homeostasis.

## 4 Materials and methods

### 4.1 Fly stocks and husbandry

Fly strains were obtained from the Bloomington *Drosophila* Stock Center, unless otherwise indicated. *Hml*-*Gal4.Delta* (48) (Bloomington #30141), *Hml-Gal4.Delta*, *UAS*-*2xeGFP* (Bloomington #30142), *UAS*-*Diap1 RNAi* (49) (Bloomington #33597), *UAS*-*hid* (50) (Bloomington #65403), *UAS*-*lexA RNAi* (Bloomington #67945 and 67947), *UAS*-*lacZ* (Bloomington #1776), *UAS-pHRed* (10), *GR1-Gal4* (gift from Trudi Schupbach, 51), *upd3-Gal4*, *UAS*-*GFP* (gift from Perrimon Lab), *UAS-upd3 RNAi* (Bloomington #32859), *draper^Δ5^* (52), *srpHemo-mCherry* (53) (Bloomington #78358), *Δupd2Δupd3* (54) (Bloomington #55729), *Δupd3* (Bloomington #55728), *UAS-Stat92E^DN^* (55), *UAS-Ced-12 RNAi* (Bloomington #28556)*, UAS-kayak RNAi* (Bloomington #33379)*, UAS-draper RNAi* (Bloomington # 67034 (56) and 36732).

Flies were maintained on cornmeal molasses food. Flies for dissection were 3–10 days old and were supplemented with yeast paste for 2 days for well-fed conditions. For protein starvation, flies were shifted to apple juice agar lacking yeast for 16–20 h prior to dissection. Flies were grown and crossed at 25°C with standard humidity and 12 hr-light/12 hr-dark light cycling.

### 4.2 Antibodies and staining

Primary antibodies: α-Hindsight (DSHB 1G9 1:20), α-cleaved Dcp-1 (Cell Signaling), α-*GFP* (Torey Pines TP401 1:1000), α-β-Gal (Promega Z3781 1:200), α-NimC1 (gift from István Andó). Secondary antibodies: goat α-rabbit AF488 (1:200), goat α-mouse AF488 (1:200), goat α-mouse Cy3 (1:200), goat α-mouse AF647 (1:200). Ovaries were dissected in 1x Phosphate Buffered Saline (PBS) and fixed in 4% Paraformaldehyde (PFA). After washing with 1x PBS + 1% Triton X-100 (PBT) to remove PFA residue, ovaries were blocked in PBANG (1x PBT, 0.5% BSA, 5% Normal Goat Serum) for 1 hour at room temperature. Primary antibodies were diluted in PBANG and incubated overnight at 4°C. The following day, ovaries were washed and incubated in secondary antibodies diluted in PBANG for 1 hour at room temperature in the dark. To visualize F-actin, Rhodamine phalloidin (1:400, Invitrogen) was added during secondary antibody incubation. Following the secondary antibody incubation, ovaries were washed in 1x PBT to remove excess antibody and were incubated in 1x PBT for 1 hour at room temperature. Ovaries were then placed in Vectashield mounting media + DAPI (Vector Labs H-1200–10) and left to sit overnight at 4°C. Following staining, ovaries were mounted on slides, and sealed with a coverslip and nail polish.

### 4.3 Whole fly fix and stain

Flies were fed with yeast paste for 1 day and then either re-fed for 1 day or protein starved for 16–20 hours on apple juice agar (57). Whole flies were then placed in 4% PFA and rotated in the dark at room temperature for 4 hours or at 4°C for 24 hours. Flies were rinsed 3x with PBS and placed in a 15% sucrose solution for 1 day and then placed in a 30% sucrose solution for 1 day. Processed flies were then placed dorsal side down in aluminum mold and frozen in Tissue-Tek O.C.T. compound. Samples were stored at -80°C until cryosectioning. Samples were cryosectioned on a Leica CM3050S cryostat at 20um and attached to slides. Slides were dried at 37°C for 1 hour and placed in -20°C for storage. Slides were then rehydrated in PBS for 10 minutes and sections were outlined using ImmEdgePen (Vector Laboratories H-4000). Samples were blocked in PBANG for 1 hour at room temperature. Primary antibody staining was done overnight at 4°C in a wet chamber. The following day, slides were washed with PBS and incubated in secondary antibody for 1–2 hours at room temperature in the dark. Slides were washed again and incubated in Hoechst 33342, trihydrochloride, trihydrate (Invitrogen H3570) (1:1000) for 10 minutes at room temperature. Slides were dried and ProLong Diamond anti-fade mounting media (Invitrogen P36961) was used to seal slides with a coverslip. Imaging was done on an Olympus BX60 upright fluorescent microscope, and images were taken at 512x512.

### 4.4 Propidium iodide staining and analysis

Flies were fed with yeast paste for 2 days and then dissected in PBS. The ovaries were then incubated in propidium iodide (Molecular Probes P-3566) (1:100) for 15 minutes at room temperature in the dark. After washing 3x in PBS, ovaries were placed in Hoechst stain (1:1000) for 10 minutes at room temperature in the dark. Ovaries were then washed 20 minutes (3x) in PBS and then mounted on slides in Voltalef oil. Ovaries were imaged immediately on the Olympus BX60 microscope and images (taken at 512x512) were processed in ImageJ. Images were measured for intensity by outlining the tissue and measuring the integrated density of the tissue. The integrated density was then divided by the tissue area to determine the intensity per μm^2^.

### 4.5 Egg chamber quantification

Flies were fed for 2 days before following DAPI staining protocol. Mounted ovaries were then imaged at 10x on Olympus BX60 upright fluorescent microscope. The number of germaria was counted as well as the number of dying mid-stage egg chambers. The percentage of dying mid-stage eggs was then calculated by dividing the number of dying eggs by the number of germaria and multiplied by 100. To quantify corpus lutea, flies were fed for 2 days before following antibody staining protocol. Mounted ovaries were imaged at 10x on Olympus BX60 upright fluorescent microscope. The number of germaria was counted as well as the number of corpus lutea. The ratio of corpus luteum was then calculated by dividing the number of corpus lutea by the number of germaria.

### 4.6 Hemocyte quantification in the oviduct

Flies were fed with yeast paste supplement before being protein starved. *srpHemo-mCherry* females or stated genotypes with NimC1 antibody staining had the whole reproductive system dissected out and fixed before following the DAPI or antibody staining protocol. It is noted that the *srpHemo* marker can label polar cells, and thus these cells were excluded from the hemocyte count. Images were taken on a Nikon Confocal and manually counted using the cell count feature on ImageJ. Z-stack images were taken at 20x and enlarged on FIJI with each positive cell labeled in each z-stack images at 10μm at a time to account for size of a hemocyte. Hemocytes were thus only counted once. Only labelled objects that followed normal hemocyte morphology and were approximately 10μm were counted. Hemocytes were counted only at the entrance of the oviduct where the ovaries connect. See **Supplementary Figure 6** for visual aid of area used in quantification.

### 4.7 Imaging and image processing

Imaging was done on either a Nikon C2 confocal or Olympus BX60 upright fluorescence microscope. FIJI-ImageJ was used for all image processing. Images were matched to threshold and channels were merged to create composite images and saved as jpgs.

### 4.8 Fecundity assay

Flies were fed for 2 days before starting the assay. Five males and five females were placed into an inverted bottle over a grape juice agar plate with yeast paste supplement. Plates were changed every day for 5 days, and the number of eggs were counted. At the end of day 5, the number of eggs were totaled and divided by the number of females.

### 4.9 Survival assay

To track survival, flies were observed every 24 hours. Flies were transferred to a new vial every three days (58). Survival is plotted as Kaplan-Meier curves using GraphPad Prism. Log-ranked Mantel Cox test was performed. Comparisons on survival between two conditions is presented as a hazard ratio (HR) that scores survival rate of a test group against survival in a referent group. Both males and females were included in the survival assay with 15 of each sex used per genotype.

### 4.10 *upd3* reporter quantification

Images of late phase midstage dying egg chambers were imported into FIJI. For each late phase egg chamber, a region of interest (ROI) containing the area of the egg chamber was manually defined and the integrated density of the tissue was measured. A healthy midstage egg chamber from the same image was also measured and the background fluorescence was subtracted from the dying midstage egg chamber. The integrated density was then divided by the tissue area of the midstage dying egg to determine the intensity per μm^2^. The *draper* line #67034 was used for knockdown of *draper*.

### 4.11 Statistics

All data (excluding hemocyte localization in cryosectioned abdomens) were graphed using GraphPad Prism 10.0.3. Egg chamber quantification, corpus luteum, fertility and fecundity assays, and necrosis analysis used ordinary one-way ANOVA. Two way ANOVA was used in **Figure 5**. STAT+ hemocytes were analyzed using unpaired t-tests. Survival assay used Kaplan-Meier (Simple Survival Analysis) test.

### 4.12 Hemocyte image segmentation, feature extraction, and spatial statistics

Images of cryosectioned abdomens containing fluorescently tagged hemocytes were first pre-processed to remove background noise using Yen’s thresholding method (59) implemented in sci-kit image (60) version 0.20.0. Pixels with intensity below the estimated threshold were set to zero. Pre-processed images were imported into QuPath version 0.3.2 (61) for performing image segmentation and feature extraction. For each image, a region of interest (ROI) was first defined manually using the brush tool to identify the boundaries of the abdomen within the image and prevent the inclusion of any tissue debris or imaging artifacts in the segmentation. A custom Groovy script was then run in the QuPath script editor to fill any annotation holes in the abdomen ROI, perform hemocyte segmentation using the Watershed algorithm, perform Delaunay triangulation of detected hemocytes, and export abdomen ROI features such as centroid and area of the abdomen, as well as detected hemocyte features such as centroid, area, pixel intensities, and cartesian coordinates. To perform positive cell detection, score compartment parameters such as channel and pixel intensity threshold were chosen based on the fluorescent tag expressed by hemocytes (green channel mean intensity, *Hml*>GFP) and the confocal laser settings used during acquisition. All the features extracted were exported as CSV files for downstream analysis. Small sections obtained from the dorsal and ventral extremities of the abdomen were excluded and only sections that were comparable in area across replicates were used in the statistical analysis to prevent any confounding arising from large differences in the area of the sections and the number of usable sections obtained per fly. Because cryosectioning small structures can affect the structural integrity of tissues by introducing minor tears, we simplified our analysis to only consider the area of the sections and disregarded any shape differences between the sections. Our final dataset after quality control included 23 sections from 8 fed flies and 31 sections from 10 starved flies. Subsequent exploratory data visualization and statistical analyses were performed in Python version 3.11.5. To obtain percentile distances from abdomen centroid, we used the distance of the farthest hemocyte as the radii of the circumcircle (Rc) encompassing the abdomen since at least one hemocyte was found close to the farthest edge of the abdomen in all sections. Concentric circles around the abdomen centroid were then constructed using radii (Ri) by splitting Rc into ten parts using the formula Rc = Ri √(i/10) where i ∈ [1,10]. Percentile distances from abdomen centroid were used to overcome differences in abdomen areas between experimental groups and replicates. Nearest neighbors G-function curves were computed using the Nearest Neighbors method implemented in sci-kit learn (62).

### 4.13 Data & code availability

Annotated images, hemocyte coordinates, scripts and notebooks used for image segmentation and statistical analysis, along with documentation, are available at https://github.com/McCallLabBU/hemocyte_recruitment_image_quantification.

## Data availability statement

The raw data supporting the conclusions of this article will be made available by the authors, without undue reservation.

## Ethics statement

The manuscript presents research on animals that do not require ethical approval for their study.

## Author contributions

AC: Conceptualization, Data curation, Formal analysis, Investigation, Methodology, Visualization, Writing – original draft, Writing – review & editing. SB: Conceptualization, Formal analysis, Investigation, Methodology, Software, Visualization, Writing – original draft, Writing – review & editing. MW: Investigation, Visualization, Writing – review & editing. SS: Conceptualization, Investigation, Visualization, Writing – review & editing. KM: Funding acquisition, Project administration, Supervision, Writing – review & editing, Conceptualization.

## Supplementary material

Supplementary Figure 1 Starvation induces hemocyte localization differences. **(A)** Distribution of Log10 area of abdomens averaged across sections per fly in well-fed and starved abdomens (two-sided Mann-Whitney U-test, p-value=0.51). **(B)** Graphical representation of the estimation of percentile distances away from the abdomen centroid. White line represents the distance of the farthest hemocyte from abdomen centroid, considered to be the radius of the largest circumcircle of the abdomen Rc. Smaller concentric circles were obtained by splitting Rc into ten equal parts. **(C)** Histogram of the number of neighbors for each hemocyte as estimated by Delaunay triangulation. **(D)** Histogram of the Log10 mean triangle area formed between hemocytes as estimated by Delaunay triangulation (Adt) as a fraction of abdomen area (Aab). **(E)** ECDF of the distances of 5 nearest neighbors for each hemocyte (G-function) in well-fed and starved abdomens. Mean ECDF of fed and starved replicates are indicated by dark lines and the 95% confidence intervals are indicated by the lighter color bands **(F)** Quantile-Quantile plot of the distribution of distances of 5 nearest neighbors of hemocytes detected in starved and well-fed abdomens, scaled by square root of abdomen area (2-sample Kolmogorov-Smirnov test p-value 0.04) **(G)** Histogram of 5 nearest neighbor distances scaled by square root of the abdomen area.

Supplementary Figure 2 Ablation of hemocytes results in a lower lifespan. Ablated flies *Hml>Diap1 RNAi* and *Hml>hid* showed a decrease in lifespan, with the average lifespan at 27 (*Hml*>*hid*) and 60 (*Hml*>*Diap RNAi*) days whereas control flies averaged 65 days. n = 7–10 females per repetition with total of > 30 flies per genotype. (** p-value < 0.003 for both ablation genotypes compared to the control).

Supplementary Figure 3 Hemocyte ablation results in an increase of midstage death. **(A)** Ablated flies *Hml*>*Diap1 RNAi* and *Hml* > *hid* showed an increase in midstage death in fed (conditioned) flies that were comparable to those of starved flies, whereas control flies had an increase of midstage death only in starved flies. **(B)** Fecundity assay of ablated and control females. Both *Hml > Diap1 RNAi* and *Hml> hid* revealed that hemocyte ablation resulted in a decrease in egg laying compared to controls. n = 5 females per replicate with total of 15 females per genotype (*** p-value < 0.0003).

Supplementary Figure 4 Follicle cells do not engulf other follicle cells in midstage death. *GR1-Gal4*>*pHRed*; *Hml*-*V5-FLAG* flies were fed and starved and the ovaries were examined. **(A)** Late phase dying egg chambers showed no pHRed fluorescence before entering the oviduct. **(A’)** pHRed channel only. **(B)** pHRed expression was seen in the entrance of the oviduct. **(B’)** pHRed channel only. Scale bar = 50μm.

Supplementary Figure 5
*Upd3* expression in knockdowns of phagocytic genes. **(A)** Graph of *updΔ3* mutants and *w^1118^* germline engulfment as death progresses. **(B)** Representative images of knockdowns (*draper RNAi* #67034) of candidate activators of *upd3*. DAPI (cyan) in top panels and *upd3-lacZ* (magenta) in bottom panels with white arrows demarking dying egg chambers.

Supplementary Figure 6 Hemocyte localization in the oviduct. Anti-NimC1 single channels from **Figure 6**. **(A)** Hemocyte localization in the oviduct of *w^1118^* starved female. **(B)** *w^1118^* fed female. **(C)** *GR1 > +* starved female. **(D)** *GR1 > upd3 RNAi* starved female. White dashed boxes highlight the area where the oviduct and ovaries connect, and quantification was done on that area. Scale bar = 100μm.
