# Supplementary figures and images for: Professional phagocytes are recruited for the clearance of obsolete nonprofessional phagocytes in the Drosophila ovary
Source: Front Immunol. 2024 Jun 27;15:1389674. doi: 10.3389/fimmu.2024.1389674 (PMC11236694; doi:10.3389/fimmu.2024.1389674)

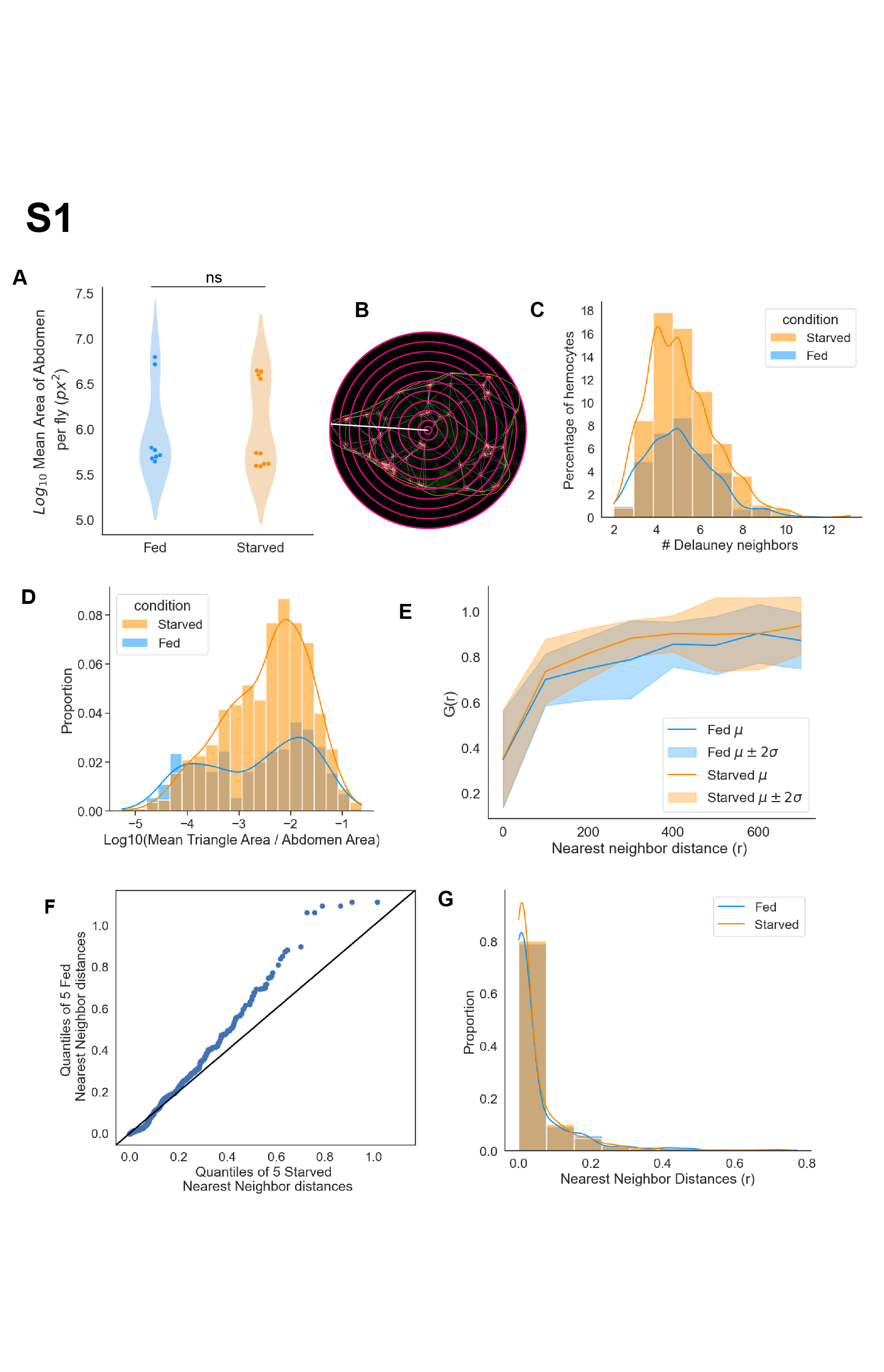

Supplement: Supplementary Figure 1 — Starvation induces hemocyte localization differences. (A) Distribution of Log10 area of abdomens averaged across sections per fly in well-fed and starved abdomens (two-sided Mann-Whitney U-test, p-value=0.51). (B) Graphical representation of the estimation of percentile distances away from the abdomen centroid. White line represents the distance of the farthest hemocyte from abdomen centroid, considered to be the radius of the largest circumcircle of the abdomen Rc. Smaller concentric circles were obtained by splitting Rc into ten equal parts. (C) Histogram of the number of neighbors for each hemocyte as estimated by Delaunay triangulation. (D) Histogram of the Log10 mean triangle area formed between hemocytes as estimated by Delaunay triangulation (Adt) as a fraction of abdomen area (Aab). (E) ECDF of the distances of 5 nearest neighbors for each hemocyte (G-function) in well-fed and starved abdomens. Mean ECDF of fed and starved replicates are indicated by dark lines and the 95% confidence intervals are indicated by the lighter color bands (F) Quantile-Quantile plot of the distribution of distances of 5 nearest neighbors of hemocytes detected in starved and well-fed abdomens, scaled by square root of abdomen area (2-sample Kolmogorov-Smirnov test p-value 0.04) (G) Histogram of 5 nearest neighbor distances scaled by square root of the abdomen area. [file Image_1.jpeg]

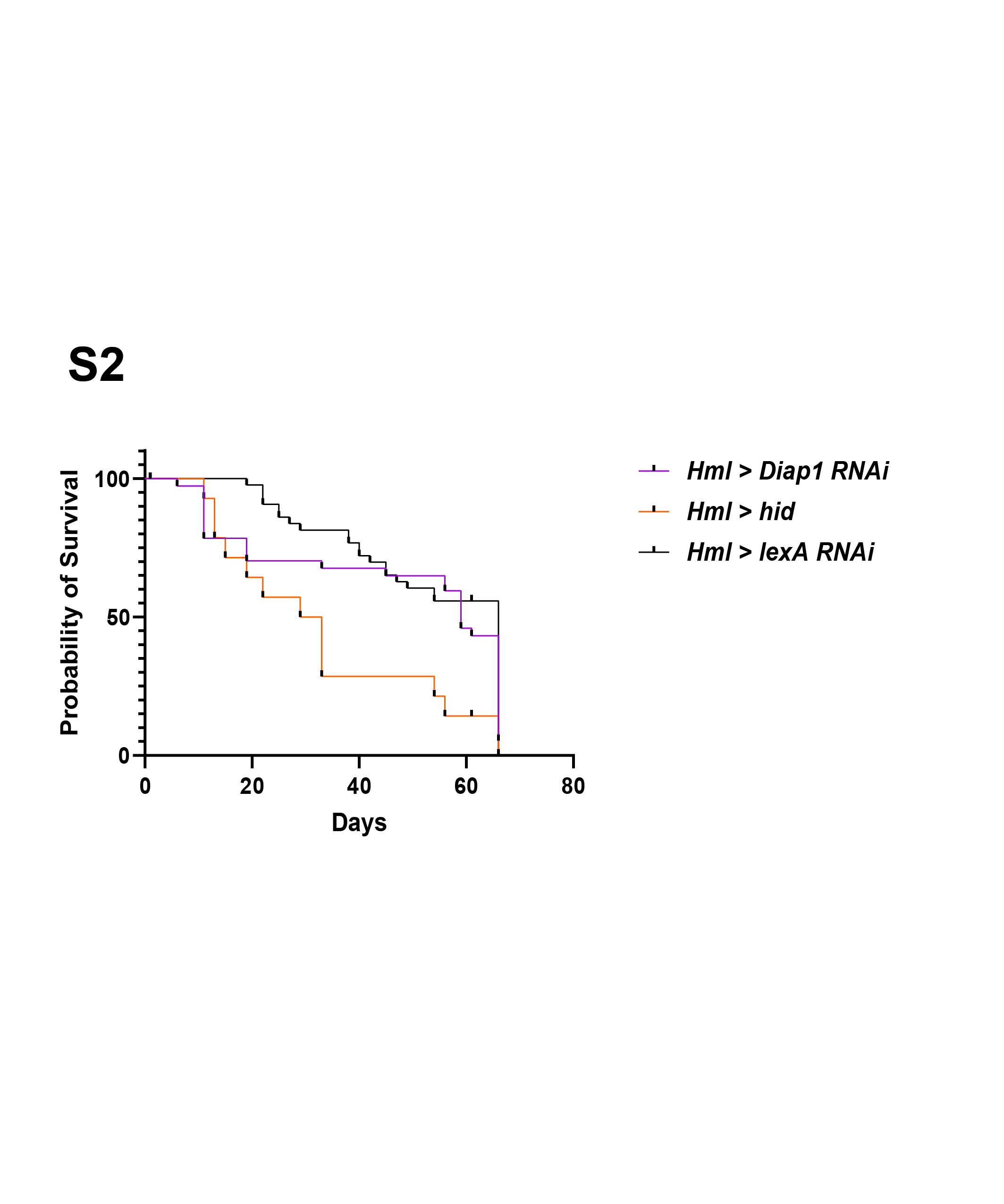

Supplement: Supplementary Figure 2 — Ablation of hemocytes results in a lower lifespan. Ablated flies Hml>Diap1 RNAi and Hml>hid showed a decrease in lifespan, with the average lifespan at 27 (Hml>hid) and 60 (Hml>Diap RNAi) days whereas control flies averaged 65 days. n = 7–10 females per repetition with total of > 30 flies per genotype. (** p-value < 0.003 for both ablation genotypes compared to the control). [file Image_2.jpeg]

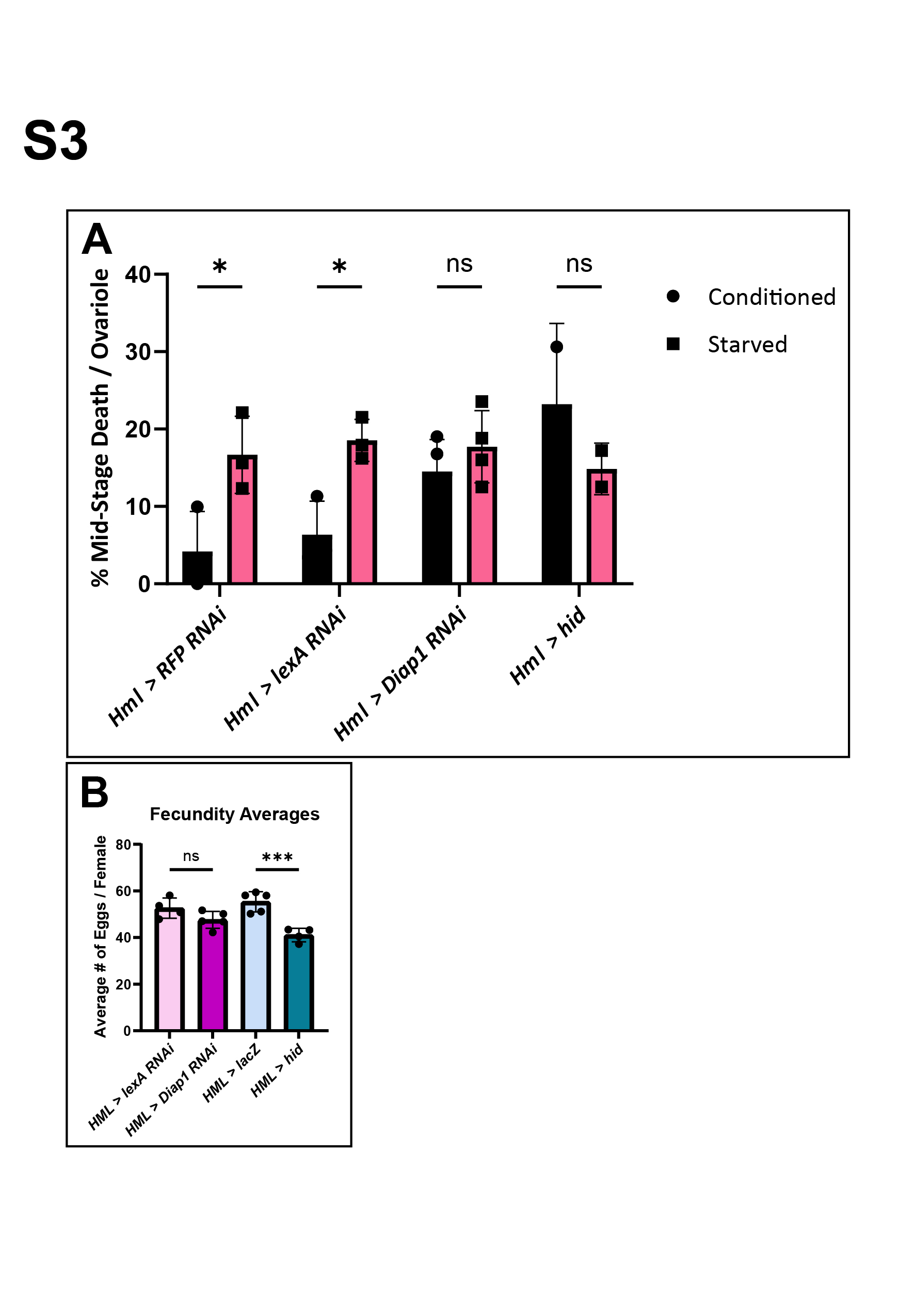

Supplement: Supplementary Figure 3 — Hemocyte ablation results in an increase of midstage death. (A) Ablated flies Hml>Diap1 RNAi and Hml > hid showed an increase in midstage death in fed (conditioned) flies that were comparable to those of starved flies, whereas control flies had an increase of midstage death only in starved flies. (B) Fecundity assay of ablated and control females. Both Hml > Diap1 RNAi and Hml> hid revealed that hemocyte ablation resulted in a decrease in egg laying compared to controls. n = 5 females per replicate with total of 15 females per genotype (*** p-value < 0.0003). [file Image_3.jpeg]

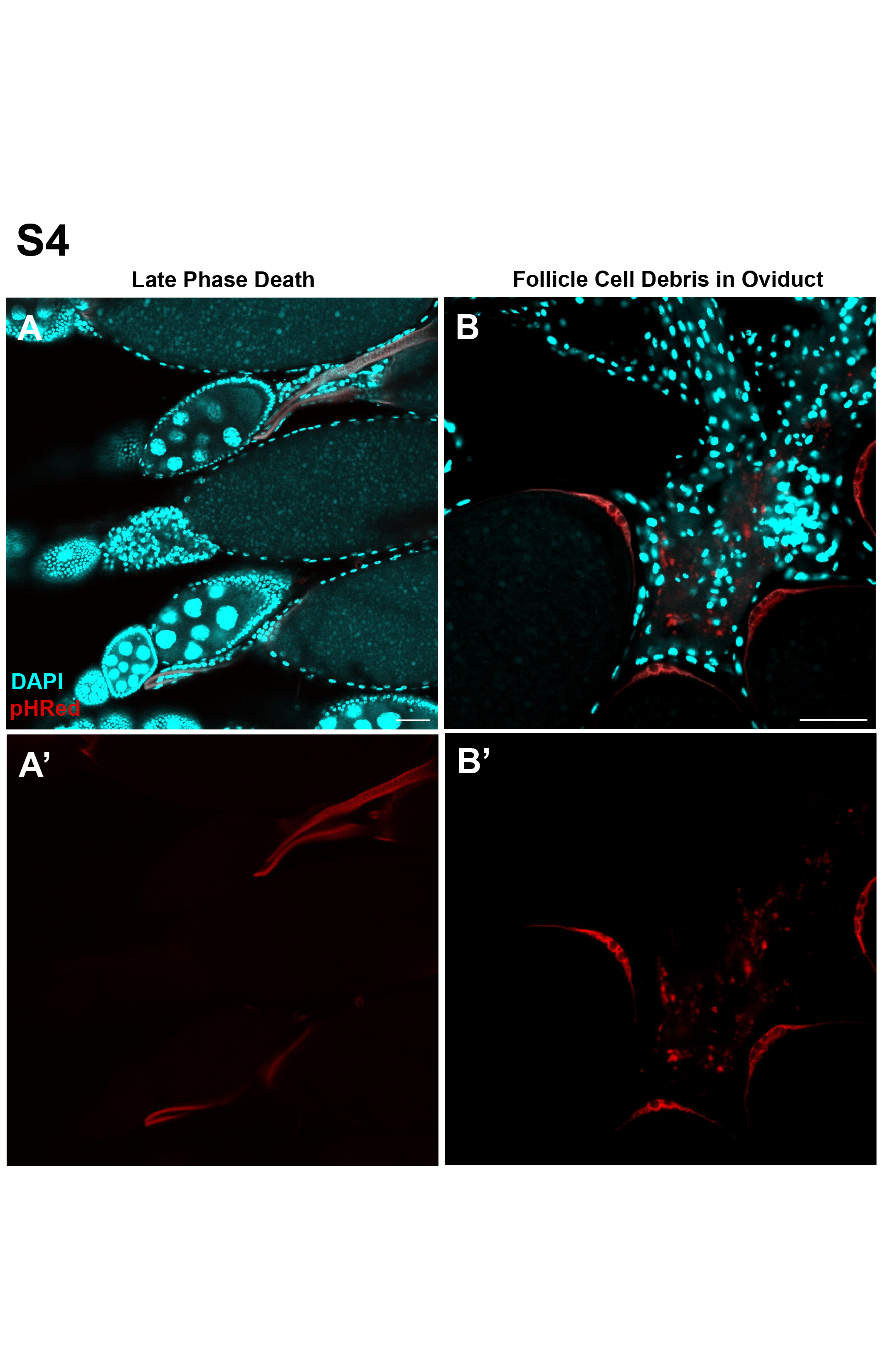

Supplement: Supplementary Figure 4 — Follicle cells do not engulf other follicle cells in midstage death. GR1-Gal4>pHRed; Hml-V5-FLAG flies were fed and starved and the ovaries were examined. (A) Late phase dying egg chambers showed no pHRed fluorescence before entering the oviduct. (A’) pHRed channel only. (B) pHRed expression was seen in the entrance of the oviduct. (B’) pHRed channel only. Scale bar = 50μm. [file Image_4.jpeg]

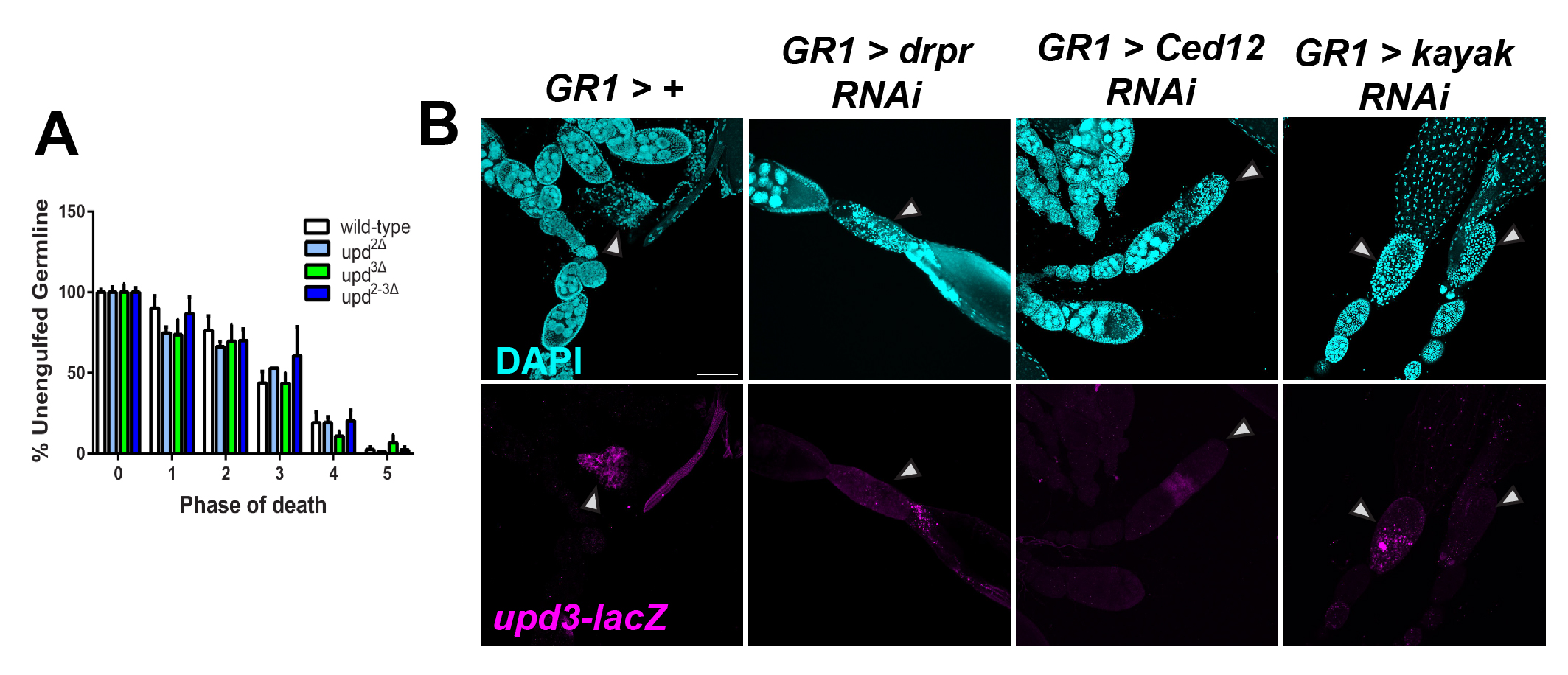

Supplement: Supplementary Figure 5 — Upd3 expression in knockdowns of phagocytic genes. (A) Graph of updΔ3 mutants and w1118 germline engulfment as death progresses. (B) Representative images of knockdowns (draper RNAi #67034) of candidate activators of upd3. DAPI (cyan) in top panels and upd3-lacZ (magenta) in bottom panels with white arrows demarking dying egg chambers. [file Image_5.jpeg]

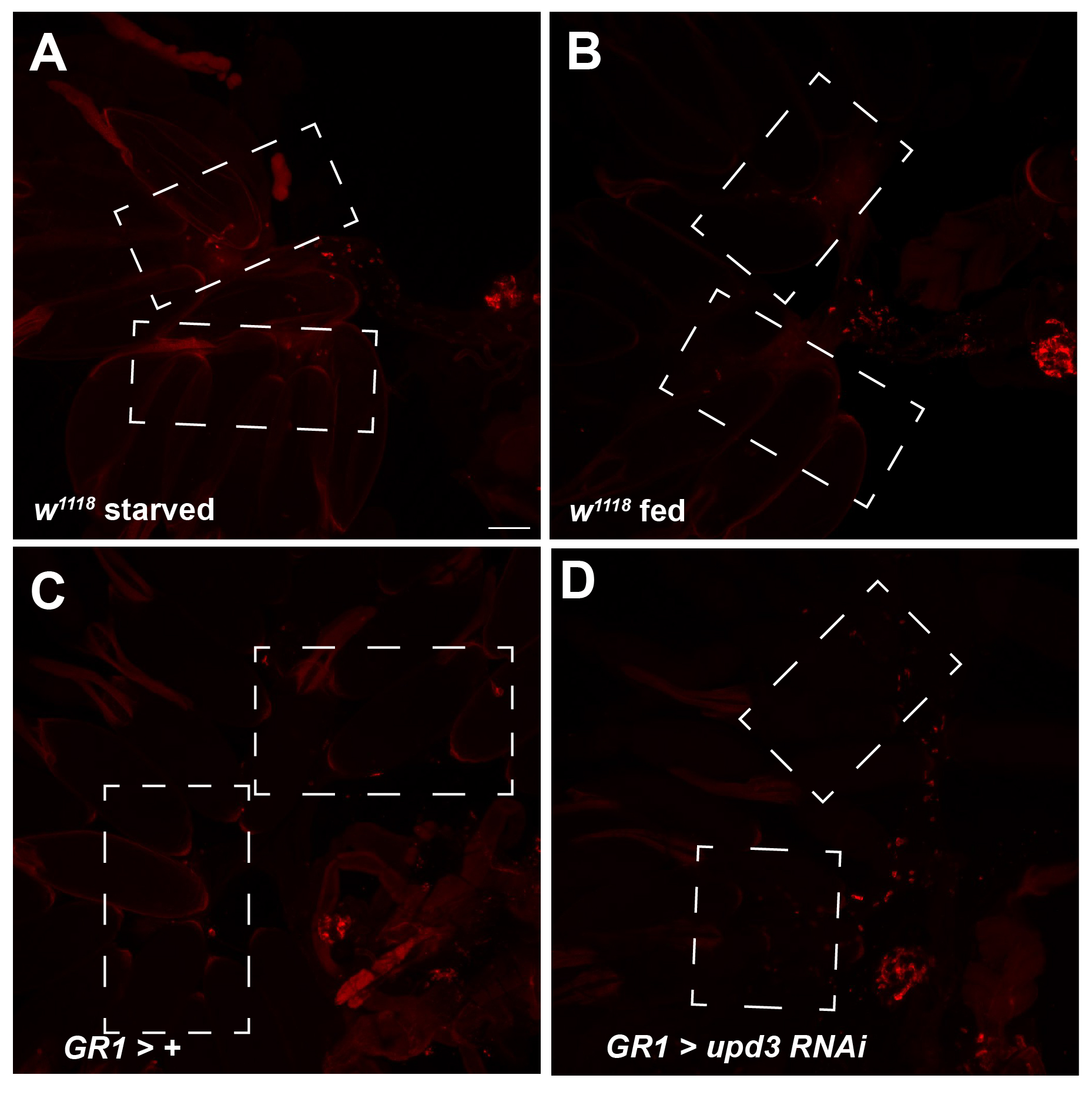

Supplement: Supplementary Figure 6 — Hemocyte localization in the oviduct. Anti-NimC1 single channels from Figure 6 . (A) Hemocyte localization in the oviduct of w1118 starved female. (B) w1118 fed female. (C) GR1 > + starved female. (D) GR1 > upd3 RNAi starved female. White dashed boxes highlight the area where the oviduct and ovaries connect, and quantification was done on that area. Scale bar = 100μm. [file Image_6.jpeg]
